# *CITED2* Mutation and methylation in children with congenital heart disease

**DOI:** 10.1186/1423-0127-21-7

**Published:** 2014-01-24

**Authors:** Min Xu, Xiaoyun Wu, Yonggang Li, Xiaofei Yang, Jihua Hu, Min Zheng, Jie Tian

**Affiliations:** 1 Department of Cardiology, Children’s Hospital of Chongqing Medical University, 136 Zhongshan Er Road, Chongqing 400014, P.R. China; 2 Department of Cardio-thoracic surgery, Children's Hospital of Chongqing Medical University, Chongqing 400014, P.R. China; 3 Ministry of Education Key Laboratory of Child Development and Disorders, Key Laboratory of Pediatrics in Chongqing, CSTC2009CA5002, Chongqing International Science and Technology Cooperation Center for Child Development and Disorders, Chongqing, P.R. China

**Keywords:** CITED2, Mutation, Methylation, Congenital heart disease

## Abstract

**Background:**

The occurrence of Congenital Heart Disease (CHD) is resulted from either genetic or environmental factors or the both. The CITED2 gene deletion or mutation is associated with the development of cardiac malformations. In this study, we have investigated the role of CITED2 gene mutation and methylation in the development of Congenital Heart Disease in pediatric patients in China.

**Results:**

We have screened 120 pediatric patients with congenital heart disease. Among these patients, 4 cases were detected to carry various CITED2 gene heterozygous mutations (c.550G > A, c.574A > G, c.573-578del6) leading correspondingly to the alterations of amino acid sequences in Gly184Ser, Ser192Gly, and Ser192fs, respectively. No CITED2 gene mutations were detected in the control group. At the same time, we found that CITED2 mutations could inhibit TFAP2c expression. In addition, we have demonstrated that abnormal CITED2 gene methylation was detected in most of the tested pediatric patients with CHD, which leads to a decrease of CITED2 transcription activities.

**Conclusions:**

Our study suggests that CITED2 gene mutations and methylation may play an important role in the development of pediatric congenital heart disease.

## Background

Congenital Heart Disease (CHD) is the most common disease in children with congenital birth defects, which is a challenge in pediatric cardiology. A body of studies has demonstrated that the congenital heart diseases are caused by either genetic or environmental factors or both. More and more gene mutations were found to be associated with CHD, such as NKX2.5, TBX5, TFAP2B, GATA-4 [1,2]. Although gene mutations are potential causes for the development of CHD [3], various environmental factors that affect mother during the pregnancy also play an important role in promoting and developing of CHD [4]. For example, epigenetic modifications and gene methylation changes in fetus during pregnancy can affect gene expression levels during the development [5-7]. Aberrant methylation in the promoter region of genes can inhibit gene transcription activities by preventing the binding of transcription factor from the target genes, which is one of the potential causes of gene silence and disease [8].

CREB-binding protein (CBP)/P300-interacting transactivator 2 is a protein with ED-rich tail that in human is encoded by the *CITED2* gene. *CITED2* expression is regulated by a variety of factors such as hypoxia, cytokines, oxidative stress, etc. [9]. There are a plurality of transcription factor binding sites in the promoter region of *CITED2* gene, such as HIF-1, AP-2, SP1 etc., which play a vital role in CITED2 expression. Three consecutive sequence of "ACGTG" in *CITED2* promoter region can maintain the stability of the combination between *CITED2* and HIF-1 [10,11]. HIF-1 consists of α, β two subtypes, in which HIF-1α is degraded under normal oxygen conditions and cannot be detected. However, HIF-1α can be detected in hypoxic conditions [12]. *CITED2* blockes HIF-1α transcriptional activity by competitively inhibiting the interaction between HIF-1α and CBP/P300, Dysfunction of HIF-1α in *CITED2*^-/-^ mice may cause the cardiac malformation [13,14]. CITED2 also functions as a transcriptional co-activator by recruiting the combination of CBP/P300 and TFAP2. Deficiencies in TFAP2 co-activation have been suggested to cause laterality defect in *CITED2*^-/-^ mice [15].

*CITED2* gene mutation was first reported by Sperling et al. in congenital heart disease [16] and the mutation could significantly diminish TFAP2c co-activation. However, the relationship between *CITED2* mutations and CHD as well as the epigenetic modification of the gene is not clear. In the present study, we have screened 120 pediatric patients with CHD and tried to reveal the role of gene mutations and epigenetic modifications in the development of CHD in children. Our study suggests that *CITED2* gene mutations and methylation may play an important role in the development of pediatric congenital heart disease.

## Methods

### Subjects

Blood samples were obtained from 120 pediatric patients (from 8 days to 18 years) and from 100 normal children with matched age. In addition, myocardial tissues were collected from 31 pediatric patients with CHD in Children's Hospital of Chongqing Medical University and 2 normal cardiac tissue samples were obtained from children died of accident (Table 1). The protocols and assays of the study was approved by the Ethics Committee in Children's Hospital Chongqing Medical University before the start of the study.

**Table 1:** Phenotypies and clinical manifestations of CHD in pediatric patients

**Congenital heart disease**	**n (%)**	**(n = 151)**
Right Atrial Isomerism	1	(0.67)
Single Atrium	1	(0.67)
Dextral Heart	2	(1.3)
Aortic Stenosis	2	(1.3)
Aortic Transection	2	(1.3)
Mirror Dextrocardia	2	(1.3)
Atrial Septal Defect with Ventricular Septal Defect	11	(7.28)
Persistent Truncus Arteriosus	3	(2.0)
Complete Transposition of Great Arteries	4	(2.65)
Pulmonary Atresia	4	(2.65)
Total Anomalous Pulmonary Venous Drainage	4	(2.65)
Single Ventricle	5	(3.31)
Pulmonary Stenosis	8	(5.3)
Double Outlet Right Ventricle	10	(6.62)
Atrial Septal Defect	24	(15.9)
Patent Ductus Arteriosus	20	(13.2)
Ventricular Septal Defect	33	(21.9)
Tetralogy of Fallot	13	(8.61)
Complete Atrioventricular Pathways	2	(1.3)

### Reagents

Blood and Tissue DNA extraction kit was purchased from Tiangen; plasmid pEGFP-C1 was purchased from Dingguo biotechnology company; LA Tag enzyme, pMD19-T simple plasmid restriction enzyme ScaI and KpnI, DNA ligase,DNA marker, Reverse transcription kits were purchased from TaKaRa; Endotoxin-free plasmid extraction kit was purchased from QIAGEN; DMEM medium, fetal bovine serum were purchased from Gibco; Lipofectamine TM 2000 was purchased from Invitrogen;SsoFastTM EvaGreen Supermix was purchased from BIO-RAD, TFAP2c primary antibody, HIF-1α, anti-β-action antibody were purchased from Bioworld;EZ DNA Methylation-Gold Kit, methylation positive control and a negative control were purchased from ZYMO;HepG2 and H9C2 cell were conserved in our laboratory.

### Detection of mutations in CITED2 gene

Blood DNA was extracted from the CHD and healthy children. *CITED2* coding region was cut into S1 and S2 to design primers. The primer sequences used in the assays are shown in Table 2. PCR amplification, and then sequencing were performed. DNA sequence was compared to the normal CITED2 coding sequence in the Genebank to reveal the mutation sites and types.

**Table 2:** Specific primers designed for the experiments

**Species**	**Gene**	**Primer sequences**	**Amplified length**
Human	CITED2	S1: Sense: 5'-AGGCTGTTAGTGGGATCTTGG -3'	559 bp
		Anti: 5'-CATGTAGTGGTT- GTGGGGGTAG -3'	
		S2: Sense: 5'-CCAGGTTTAACAACTCCCAGTTC-3'	
		Anti: 5'-CCACAAGATTAAGCAGTTTGCC-3'	
		BSP: Sense: 5'-GGTGGGGTAGATTTAGTTTGAGG-3'	350 bp
		Anti: 5'-ACTTTAACCACAATTAATATAAACA TTTC -3'	
		MSP: MF: 5’-CGCGTGGTGTTATACGGGACG -3’	199 bp
		MR: 5’-ACAAAACCTCCCTCCGAACT -3’	
		UF: 5’-TGTGTGGTGTTATATGGGATG-3’	
		UR: 5’-ACAAAACCTCCCTCCAAACT-3’	
	TFAP2c	Sense: 5'-AAATCCTTCTCCACCGCACAGACT-3'	100 bp
		Anti: 5'-TGATGCAGAACCAGTGAAGGCTCT-3'	
	HIF-1α	Sense: 5'-CGTTCCTTCGATCAGTTGTC-3'	143 bp
		Anti: 5'-TCAGTGGTGGCAGTGGTAGT-3'	
	β-actin	Sense: 5'-CATGGGTCAGAAGGATTCCTATGTG-3'	116 bp
		Anti: 5'-ATTTTCTCCATGTCGTCCCAGTTG-3'	
Rats	TFAP2c	Sense: 5'-GGATTTAACTGGCGACTAT-3'	79 bp
		Anti: 5'-CCTCTTCATACTTGACATTATC-3'	
	HIF-1α	Sense: 5'-CTGCCACCACTGATGAAT-3'	128 bp
		Anti: 5'-ACTGTATGCTGATGCCTTAG-3'	
	β-actin	Sense: 5'- TATGGAATCCTGTGGCATC-3'	87 bp
		Anti: 5'-GTGTTGGCATAGAGGTCTT-3'	

### Construct CITED2 mutant and wild-type recombinant plasmid

CITED2 (c.573-578del6) and normal PCR purification products were connected with pMD19-T simple plasmid. Then they were transformed into competent E. coli DH5α and the positive clones were detected. Again, the positive clones were selected and sequenced.

### Transfection of plasmids into the cells

Human hepatoma cell line HepG2 and Rats myocardial cell lines H9C2 were cultured in DMEM medium containing 10% fetal bovine serum. The medium was changed regularly. Recombinant plasmids were transfected into HepG2 and H9C2 using Lipofectamine™ 2000. The transfection efficiency of pEGFP-C1-wtCITED2 and pEGFP-C1-mtCITED2 were observed by fluorescence microscopy. The expression of CITED2 was analyzed with Western blotting. Experimental groups included: Empty vector group, Untransfected group, Mutation group and Wild-type control group.

### Detection of TFAP2c and HIF-1α mRNA expression by Q-PCR

After transfection, the cells were cultured in the different conditions (21% O_2_, 74% N_2_, 5% CO_2_ at 37°C or 1% O_2_, 94% N_2_, 5% CO_2_ at 37°C). RNA was extracted after 24 h and reversely transcribed into cDNA. HIF-1α and TFAP2c was amplified by SsoFast™ EvaGreen Supermix. The sequences of the primers used in the assays are shown in Table 2. Expression differences of TFAP2c and HIF-1α mRNA was compared among the four assay groups. Each experiment was repeated at least three times.

### Detection of TFAP2c and HIF-1α protein levels by Western blotting

After transfecting, cells were respectively cultured in the condition of 37°C, 21% O_2_, 74% N_2_, 5% CO_2_ and 37°C, 1% O_2_, 94% N_2_, 5% CO_2_. Proteins were collected after 48 h and boiled with buffer for 5 min and then separated by SDS-PAGE gel electrophoresis. Transferring protein onto PVDF and blocking 1 h with 5% skim milk. The membranes was incubated at 4°C overnight after hatching TFAP2c and HIF-1α antibodies. The PVDF was washed three times in PBST. TFAP2c and HIF-1α protein levels were compared among the four different groups. At least three independent experiments were performed.

### Detection of methylation in CITED2 gene promoter region (CpG island)

Genomic DNA was extracted from myocardial tissues of CHD and control groups and treated with bisulfite modification using an EZ DNA Methylation-Gold Kit. This treatment converts unmethylated cytosines to uracils, while methylated cytosines unchanged. CITED2 (-10 bp ~ -360 bp) region was detected by Bisulfite-PCR sequencing (BSP). The methylation sites were identified after PCR amplification of the cloning and sequencing. The methylation on CITED2 (-971 bp ~ -1171 bp) was detected using a methylation- specific PCR (MSP) assay. The methylated or unmethylated samples was analyzed by 2% agarose gel electrophoresis.

### Detection of CITED2 mRNA expression by Q-PCR

RNA was extracted from the methylated and control groups and reversely transcribed into cDNA. The transcriptional expression of *CITED2* gene was detected and compared. β-actin was used as an internal control.

CITED2: Sense: 5'-TTCCCTCACTTTCTCCAGTGCTCA-3 antisense: 5'-ATGAAGCGAGATGGCAGTTTGTGC-3', Length:191 bp; β-actin: Sense: 5'-CATGGGTCAGAAGGATTCCTATGTG-3' antisense: 5'-ATTTTCTCCATGTCGTCCCAGTTG-3', length:116 bp.

### Statistical analysis

Statistical analysis was performed using student's t-test or Fish's exact test and *X*^*2*^*test*. The obtained P values are indicated in the text. All data were analyzed with SPSS.17.0 software,

## Results

### Mutations detected in CITED2 coding region

4 heterozygous mutations were detected among 120 patients with congenital heart disease, Including 2 cases of point mutations and 2 cases of deletion mutants. These mutations lead to corresponding amino acid variations in CITED2 protein. However no mutation were detected in samples from the 100 normal controls (Table 3).

**Table 3:** CITED2 gene mutations detected in pediatric patients with CHD

**Nucleotide**	**Amino acid**	**Number**	**Phenotype**
c.550G > A	p.Gly184Ser	1	Mirror dextrocardia, right aortic arch, tetralogy of Fallot
c.574A > G	p.Ser192Gly	1	Aortic stenosis
c.573-578del6	p.Ser192fs	2	Aortic stenosis and pulmonary valve stenosis; ventricular septal defect and atrial septal defect

### Measurement of TFAP2c and HIF-1α mRNA expressions

**Figure 1:**
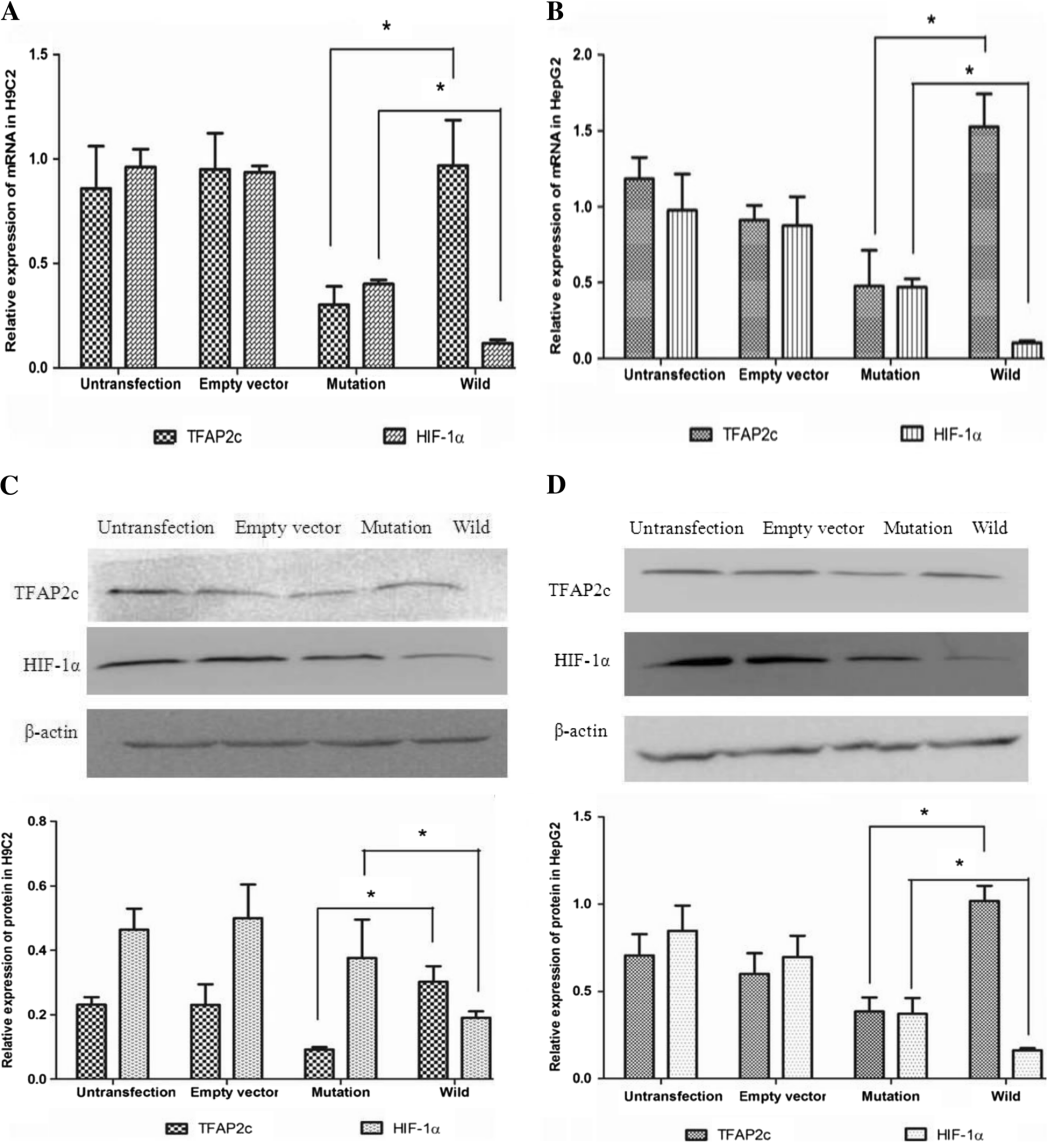
**Expression of TFAP2c, HIF-1α mRNA and protein after the transfection of CITED2 wild-type and mutant recombinant plasmids. A**: Q-PCR analysis showed TFAP2c mRNA expression levels were reduced significantly in mutant group comparing to the wild-type group in H9C2 (*P < 0.05). By contrast, HIF-1α mRNA expression levels were higher in the mutant group than that in wild- type group (*P < 0.05). **B**: Q-PCR analysis showed TFAP2c mRNA expression levels were reduced significantly in mutant group comparing to the wild-type group in HepG2 (*P < 0.05). By contrast, HIF-1α mRNA expression levels were higher in the mutant group than that in wild- type group (*P < 0.05). **C**: Western-blotting data demonstrated that TFAP2c protein concentrations were significantly lower in the mutant group comparing to the wild-type control group in H9C2 (*P < 0.05). But the concentrations of HIF-1α protein was higher in the mutant group than that of the wild-type control group (*P < 0.05). **D**: Western-blotting data demonstrated that TFAP2c protein concentrations were significantly lower in the mutant group comparing to the wild-type control group in HepG2 (*P < 0.05). But the concentrations of HIF-1α protein was higher in the mutant group than that of the wild-type control group (*P < 0.05).

CITED2 wild-type and mutant recombinant plasmids were constructed successfully. By comparison to the wild-type plasmid pEGFP-C1-wtCITED2, the recombinant mutant plasmid pEGFP-C1-mtCITED2 could be identified by the absence of six bases based on sequencing. TFAP2c mRNA expression levels were reduced significantly in mutant group comparing to the wild-type group in H9C2 (*P < 0.05*). The empty vector group and untransfected group showed no statistical difference (*P > 0.05*). By contrast, HIF-1α mRNA expression levels were higher in the mutant group than that in wild-type group (*P < 0.05*) (Figure 1A). Similarly, in HepG2 cells, we aslo found the same results, TFAP2c mRNA expression levels were reduced in mutant group comparing to the wild-type group, But HIF-1α mRNA expression levels were higher in the mutant group than that in wild-type group (*P < 0.05*) ( Figure 1B).

### Determination of protein expression levels of TFAP2c and HIF-1α

The concentrations of TFAP2c protein were reduced in the mutant group comparing to that of the empty vector group in H9C2 (*P < 0.05*). TFAP2c protein concentrations were significantly lower in the mutant group comparing to the wild-type control group (*P < 0.05*). The empty vector group and untransfected group showed no significant difference (*P > 0.05*). Similarly to the mRNA data, the concentrations of HIF-1α protein was higher in the mutant group than that of the wild-type control group (*P < 0.05*) (Figure 1C). Similarly, in HepG2 cells, It shows the same results, TFAP2c protein concentrations were lower in the mutant group comparing to the wild-type control group (*P < 0.05*), but the concentrations of HIF-1α protein was higher in the mutant group than that of the wild-type control group (*P < 0.05*) (Figure 1D).

### Detection of methylation in CITED2 promoter region

**Figure 2:**
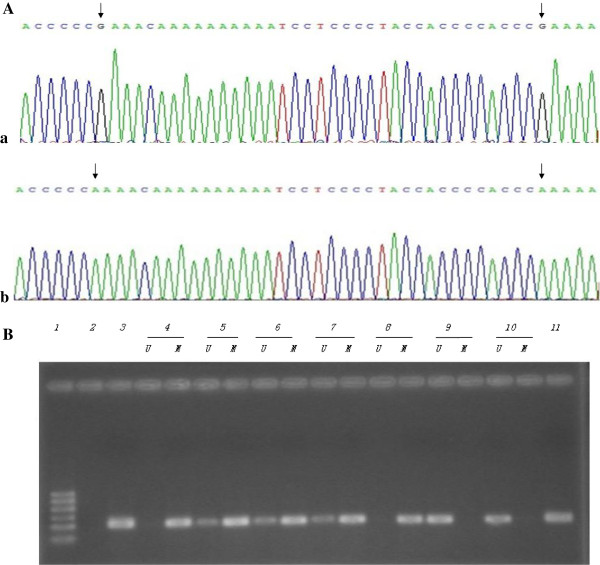
**The clone sequencing and Electrophoresis by BSP and MSP. A**. The clone sequencing of reverse complementary chain in CHD and control group by BSP. **a**: It shows the methylated sequence in CHD group, arrow represent methylated sites; **b**: The corresponding non-methylated sequences in control group, arrow represent unmethylated sites. **B**. It shows the result of Electrophoresis by MSP. it demonstrates that Methylated strip exsits in the samples with CHD,but in the control group it shows the Unmethylated strip. It demonstrates that CITED2 methylation exsits in CHD group. 1: DNA Marker 100-600 bp, 2: Blank control (ddH2O), 3: Methylated positive control, 11: Negative control, 4-8: Methylated samples in congenital heart disease, 9-10: Unmethylated samples in the control group, M: methylated strip, U: unmethylated strip.

**Figure 3:**
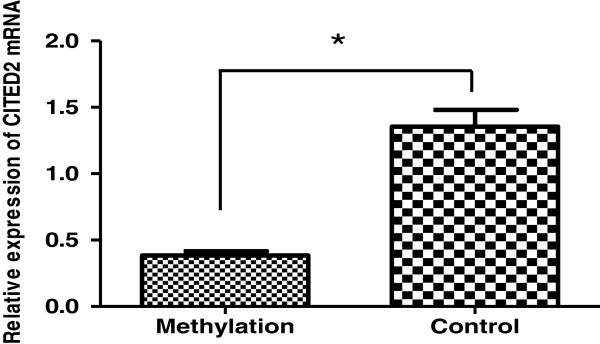
**Expression of CITED2 mRNA in CITED2 methylated group and control group.** It shows that CITED2 expression levels were significantly decreased in samples with methylation in CITED2 promoter region compared to that of the samples without methylation in CITED2 gene (*P < 0.05).

Two methods were used to detect the methylation in gene promoter region. One was to use bisulfate-PCR primers (BSP) and the other was to use methylation-specific PCR primers (MSP). Both primers were described in Materials and Methods. The sequencing data from BSP showed that methylation was detected in 10 cases out of 12 patient samples tested (83% of methylation positive rate). The samples from the normal control group were small with only two cases. However, no methylation was detected in the control samples (Figure 2A). The MSP showed that the methylation was detected in 16 cases out of 19 patients with CHD (84% of methylation-positive rate). No methylation was detected using this method in 2 normal control samples (Figure 2B). It was also found that CITED2 expression levels were significantly decreased in samples with methylation in CITED2 promoter region compared to that of the samples without methylation in CITED2 (P < 0.05) (Figure 3).

## Discussion

*CITED2* gene is one important member of the CITED family. It is located in chromosome 6q23.3 and contains three exons and two introns, encoding a protein with 270 amino acids. The amino acid sequence of CITED2 protein is characterized by three conserved regions (CR1, CR2 and CR3). CR2 is a conserved region consisting of 32 amino acids, which binds with CH1 domain of CBP/P300. A unique serine-glycine-rich region (SGJ) is located in its carboxyl terminus. CITED2 gene has about 3 kb of CpG islands in its promoter region and coding region that may be modified by methylation [10]. In this study we have detected 4 various mutations in *CITED2* coding region (c.550G > A, c.574A > G, c.573-578del6) in blood samples from pediatric patients with CHD. All of these mutations are clustered in the SGJ area. The different mutations are associated with different phenotypes of CHD in pediatric patients, and a same mutation causes different CHD phenotypes. So gene mutation along can not well explain the occurrence of CHD, suggesting that other factors play roles in the development of CHD. In this study, we have demonstrated that methylation levels of certain important genes, such as CITED2, are associated as well with the development of the CHD.

Previous studies have shown that CITED2 plays an important role in the heart development. Lack of CITED2 in embryos can cause an abnormal heart ring formation, as well as various cardiac malformations including artial and ventricular septal defects, transposition of great arteries, double outlet right ventricle, tetralogy of fallot and so on [17]. Heart-specific knockout of *CITED2* in mice leads to a ventricular septal defect and ventricular wall thinning, as well as abnormal angiogenesis, suggesting that CITED2 plays a vital role in the growth and development of ventricular muscles and ventricular septal coronary vessels [18]. CITED2 can act as a transcription cofactor assisting various transcription factors to modulate a normal development of the heart. CITED2-/- embryos can down-regulate Nodal, Lefty2 and Pitx2 expression in the left side plate mesoderm, which hampers the formation of the normal axis of the mouse heart [19]. CITED2 and HIF-1α can competitively bind on CH1 domain of CBP/P300. So CITED2 has been proposed to be a negative-feedbacked inhibitor under a hypoxic environment to limit HIF-1α overexpression. This negative feedback action is diminished when *CITED2* gene is deleted or downregulated, which leads to an overexpression of HIF-1α [20-23]. Our study has further confirmed that CITED2 can, indeed, inhibit HIF-1α expression since this inhibition is diminished by *CITED2* mutations detected in pediatric patients with CHD. As a consequence, the HIF-1α expression is enhanced in pediatric CHD patients with *CITED2* mutations. The overexpression of HIF-1α might bring the following events that are associated with the development of CHD: 1) HIF-1α can suppress cell apoptosis in cardiac outflow tract. Cell apoptosis will be inhibited because of HIF-1α overexpression, which hinders the development of cardiac outflow tract. 2) HIF-1α overexpression can increase the expression of its target gene VEGF, which blocks the development of vasculature and leads to myocardial ischemia and hypoxia. 3) HIF-1α overexpression inhibits migration and transition of neural crest cell, which hinders the formation of a normal heart.

The CR3 binding domain of CITED2 can bind with TFAP2, and its CR2 binding domain can bind CBP/P300, So CITED2 as a bridge between TFAP2 and CBP/P300 can activate the transcription activity of TFAP2. Lower TFAP2 transcriptional activity in CITED2-/- mice confirmed this view [24]. TFAP2c and TFAP2a can interact with CITED2, especially TFAP2c [25]. In our study the connection between TFAP2c and CBP/P300 weaked owing to CITED2 mutation, so there is a reduction in TFAP2c. Abnomal expression of TFAP2c can limit the development of cardiac neural rest. TFAP2c low expression further affect transcriptional regulator Pitx2 expression, which hamper the formation of the normal axis of the heart.

Methylation is a process that transfers –CH3 to the specific base. Gene methylation plays a critical role in cell proliferation, differentiation and apoptosis. Aberrant methylation was mainly induced by environmental factors. And the incidence of congenital heart disease is closely associated with environmental factors during the pregnancy. Various factors during the pregnancy, such as ethanol, riboflavin and folic acid, can induce gene methylations in vivo [26-30]. Studies have shown that maternal high-fat diet combined with *CITED2* gene deletion can increase penetrance of the heart abnormality body axis [31]. A close correlation has been discovered between congenital heart disease with the *CITED2* gene deletion and environmental alteration. Zhu et al. reported that several abnormally methylated bases were found in ventricular septal defects and NOX5 hypermethylation in CpG island can inhibit its expression [32]. Our study confirms that methylation of *CITED2* gene promoter region is observed in pediatric patients with CHD and the methylation can decrease *CITED2* transcription activity.

## Conclusions

In conclusion, our study discovers the *CITED2* gene mutations as well as CITED2 gene promoter region methylation in pediatric patients with CHD, which affects transcriptional activity of TFAP2c and HIF-1α. The latter are closely associated with the heart development. Our data suggest that both gene mutations and epigenetic modifications play a role in the development of CHD.

## Consent

Written informed consent was obtained from the patient for the publication of this report and any accompanying images.

## Competing interests

The authors declare that they have no competing interests.

## Authors’ contribution

XYW, JT, XFY and JHH designed research planning. YGL participated in the patient data collection. MX, XFY, JHH and MZ performed the experiments. XYW, MX, XFY, JHH and MZ analyzed the data and writing the manuscript. XYW was a responsible author and gave final approval of the version to be published. All authors read and approved the final manuscript.
